# Bipolar Disorder in the Elderly: Clinical Insights and Therapeutic Challenges

**DOI:** 10.1192/j.eurpsy.2024.431

**Published:** 2024-08-27

**Authors:** A. F. Borges, V. Domingues

**Affiliations:** Serviço de Psiquiatria e Saúde Mental, Centro Hospitalar de Leiria, Leiria, Portugal

## Abstract

**Introduction:**

Bipolar Disorder (BD) is a mood disorder characterized by recurrent episodes of mania, hypomania, and depression. While it often manifests in early adulthood, it can persist or emerge in later life, posing unique diagnostic and therapeutic challenges in the elderly population. This abstract explores the clinical aspects, diagnostic intricacies, and therapeutic considerations of BD in older adults.

**Objectives:**

This study aims to shed light on the epidemiology and clinical presentation of BD in the elderly, discuss the diagnostic challenges, and address the complexities of treatment and management in this age group.

**Methods:**

A comprehensive review of the literature was conducted, encompassing epidemiological studies, clinical trials, case reports, and expert guidelines from the past decade. The search was performed using medical databases such as PubMed and Medline.

**Results:**

BD in the elderly presents with a range of clinical complexities that differentiate it from presentations in younger adults. These complexities include atypical features as elderly individuals may exhibit less overt manic or hypomanic symptoms, resembling irritability rather than euphoria; depressive episodes can be more prevalent and prolonged, leading to potential misdiagnosis as unipolar depression; medical comorbidities: older adults with BD often have more medical conditions, complicating treatment; cognitive impairment: cognitive decline, including mild cognitive impairment and dementia, is common and distinguishing it from neurodegenerative conditions requires specialized assessment; mixed episodes, in older adults may experience mixed episodes, requiring intensive treatment; diagnostic challenges: overlapping symptoms with other disorders make accurate diagnosis challenging. Treatment includes mood stabilizers like lithium, valproate, or lamotrigine, and atypical antipsychotics like quetiapine or aripiprazole. Treatment response varies, requiring consideration of age-related pharmacokinetics, pharmacodynamics, and drug interactions. Non-pharmacological interventions, including psychoeducation, tailored cognitive-behavioural therapies, and psychosocial support, are essential.

**Conclusions:**

In summary, BD in the elderly demands a customized, multidisciplinary approach to navigate diagnostic complexities and optimize treatment, considering comorbidities and cognitive factors. Enhanced clinical awareness and holistic care are essential for effective management in this population.

**Disclosure of Interest:**

None Declared

